# Inflammatory Cytokines and Physical Activity in Multiple Sclerosis

**DOI:** 10.1155/2014/151572

**Published:** 2014-01-27

**Authors:** Margarida Florindo

**Affiliations:** Department of Physiotherapy, Portuguese Red Cross Superior Health School, Avenida de Ceuta, Edifício Urbiceuta, 1300-125 Lisboa, Portugal

## Abstract

*Background*. Besides the functional benefits, physical activity triggers a hormonal pattern of immunologic responses with an anti-inflammatory effect in individuals who suffer from multiple sclerosis. *Purpose*. To analyze the influence of physical activity on multiple sclerosis and identify the intensity threshold which triggers the anti-inflammatory physiological mechanism. *Methodology*. A systematic review was made on the databases Medline, PubMed, ScienceDirect, PloS, PEDro, and Web of Science. Studies from references of retrieved articles were also collected. The criteria included studies published in English and random studies referred to the inflammatory process, connected with physical activity in individuals with multiple sclerosis. The studies were methodologically analyzed by two reviewers according to PEDro scale. *Results and Discussion*. Five random control trial studies were identified. The results revealed that with physical activity there seems to have a modulation on anti-inflammatory cytokines which improve physical and cardiorespiratory performance. More investigation is required. *Conclusions*. Physical activity influences the quality of life and it seems to stimulate the presence of anti-inflammatory cytokines. With light physical activity the cellular activity is lower, while with moderate activity there seems to have more capacity to help in the resolution of an inflammatory situation.

## 1. Introduction

Multiple sclerosis (MS) is a demyelinating autoimmune disease, with an unclear etiological mechanism, and characterized by lymphocyte infiltration, several inflammatory patterns, and axonal loss [1, 2]. Affecting over two million individuals in the world, MS is the most common cause of disability in young adults, interfering with functional mobility and autonomy [3].

Physical activity has been referred to as a factor that increases the immunological cells circulation, triggering a physiological mechanism and contributing to the plasticity and the neuroprotection of the nervous system [4, 5]. The physical activity adequacy to the stage of the disease, during a physiotherapy intervention, allows a strengthening of immunity, together with the development of movement, cognitive and functional independence abilities.

During the last years, several studies have been made on the physiological benefits that physical activity causes in the immune system. When physical activity is present, the human body activates a hormonal pattern for an immunologic response [6, 7], which allows an immunologic homeostatic environment balance, by the inflammatory markers activity [6–8] and by influencing the helper T lymphocytes (Th), resulting in beneficial effects on cardiorespiratory function and physical capabilities and in the individual quality of life [2, 8]. Physiotherapy through physical activity has an effect on the immune system such as facilitating the cells flux, the identification of the antigen, and the reparation of damaged tissue [9–11]. Physical activity is considered a physiological disturbance which influences the leukocytes mobility and function, contributing to angiogenesis and tissue repair through the influence of the growth factors and cytokines. Some studies assume that cytokines and other peptides that are produced, expressed, and released by muscular fibers have endocrine effects and facilitate the production of anti-inflammatory components [12].

The purpose of this review is to analyze the influence of physical activity on multiple sclerosis metabolism, identifying the intensity threshold which triggers the anti-inflammatory physiological mechanism.

## 2. Methodology

A systematic review was made through electronic searches on the databases Medline, PubMed, ScienceDirect, PloS, PEDro, and Web of Science, using the keywords multiple sclerosis, anti-inflammatory, cytokines, exercise, physical activity, and physiotherapy. One thousand and twenty-one papers were found during research done up to the year 2012. After a survey of titles or abstracts seventy-nine of these papers were compiled, where the main subject was related to the process of autoimmune metabolism and the role of physical activity in MS individuals.

In a second phase, the paper's bibliography used in the first phase was revised and twenty-four more papers concerning the study thematic were included. One hundred and three studies were collected as a whole.

Included in the criteria were studies published in the English language and random controlled trials (RCT). According to the theme aim and specificity, fifteen RCTs were identified and ten of them were excluded, as they did not establish a connection between physical activity and the inflammatory cytokines evaluation in MS individuals or because they had not been tested on humans. Five studies were included which fulfilled the criteria of the systematic review. The included studies were methodologically analyzed by two reviewers (Table 1), who evaluated them through seven to eight steps according to PEDro scale. The punctuation was only attributed to each criterion when it was clearly present [13].

## 3. Results

After the parameters definition (sample, inflammatory markers, physical activity duration, frequency and type, and results), a table was elaborated to better read the obtained results (Table 2), and the selected papers were analyzed afterwards.

The training performed during the studies lasted thirty minutes and the kind of training alternated between aerobics and resistance training. The report of alterations in the cytokine concentration seems to be more evident after the second or third day of physical activity and the effects of repeated physical activity increased significantly the concentration of inflammatory cells, during the first period (minimum one hour) of a moderate intensity exercise (65% of maximal oxygen consumption—VO_2max_). The studies provided information about the epinephrine influence in the mobilization of T- and B-cell subsets which, increased by physical activity [2–14].

Table 2 presents the results of the studies classified in functional, endocrine, and inflammatory parameters and also the conclusions of each study.

## 4. Discussion

In MS the regular practice of physical activities seems to revert the chronic inflammation, promoting the decrease of proinflammatory cytokines TNF-*α* and IFN-*γ*, and the increase of IL-6 and IL-10 concentration [15–17]. Besides the participation in endocrine and anti-inflammatory mechanisms, global benefits in the quality of life were identified, such as the increase of cardiac and respiratory functions, weight loss, balance, strength, and cognitive functions. Several hormones are susceptible to muscular stress and were affected by the activity type, volume, intensity, and muscular groups involved and by the duration of resting pauses [18, 19].

Some reports refer to the intensive and extended exercise effects mainly in Th1 and Th2 cells, keeping up the segregation and modulation of other cytokines in inflamed tissues in healthy individuals [2, 20], assuming that the stress produced by exercise may induce changes in cellular activity, and this seems to occur in MS individuals as well [5, 12, 14, 21].

The demyelinization process in MS is characterized by oligodendrocytes damage, leading to the appearance of demyelinization zones with random proliferation of astrocytes in the injury area which fills the damaged tissue area and forming an astrocytic scar which interrupts the action potential. Some other axons may temporarily survive, and there is some remyelinization by the growth of oligodendrocyte precursor cells, which remain in the adjacent areas, but this reparation process generally does not remain for a long time. It seems that the autoimmune alteration in MS is caused by T-cells of Th1 type and Th17 type (IFN-*γ*, TNF-*α*, and IL-17), while the anti-inflammatory cytokines of Th2 type (IL-6 and IL-10) have been associated with remission periods and the recovery from the disease [2, 22–29].

As we could observe in Table 2, the studies without the association immunotherapy exercise [2, 8, 14, 22] and the results obtained for IFN-*γ* had significant lower value. In the only study considered that included immunotherapy [12] the IFN-*γ* was not assessed; therefore, it is not clear that the association exercise immunotherapy presents results that are different from those ones without immunotherapy. In our opinion, it does not exclude the influence of the exercise itself.

The effects of physical activity in cardiorespiratory function were evaluated by VO_2max_ and by heart frequency, with improvement of both in every group that performed the activity. According to recent studies, fatigue is associated with the quality of life, while the cardiorespiratory function or resistance is associated with fatigue [30–32]. Within the values concerning the quality of life, the fatigue levels were particularly evaluated, and a significant improvement was clearly identified.

In a first analysis, it was verified that the authors who have studied the IL-6 did not find alterations among the groups as there was a similar increase in all the individuals before, during, or after the acute activity [8, 12, 22]. Another study based on healthy individuals also referred to a higher response of IL-6 concentration during intermittent high-intensity exercise, comparing with a continuous moderate-intensity exercise [33]. The IL-6 production is subject to the activity and to the alternation between exercise and rest periods. One of the studies [8] suggested that the IL-6 inflammatory markers answered in the same way to the physical activity, whether the individuals were healthy or had MS.

The inflammatory markers of IL-6 in MS and healthy individuals seem to respond similarly to physical activity. During the skeletal muscle activity, the release of IL-6 increased significantly and probably influenced the inflammation process, oxidative stress, and synaptic activity [7, 21].

The effects of physical activity in sIL-6r were evaluated only in one of the studies [12] and tended to be high after moderated/intense activity (75% VO_2max_) in MS individuals. The significant increase of IL-6 may stimulate the production of it soluble receptor (sIL-6r) that when differing it has blockage functions to Th2, induces the proliferation of TNF-*α*, and regulates the cellular activity in blood-brain barrier [20]. There was a similar answer in healthy individuals when they were exposed to an intense and extended physical activity (e.g., high competition athletes and marathon runners). The tendency of sIL-6r to become high after physical activity, in MS, corresponds exactly to the results of healthy individuals, after an extended activity [12, 34].

The IL-17 was analyzed in one study with similar values at the beginning and with a significant decrease at the group that performed the physical activity [2]. The IL-17 cytokine (related to the lesion activity) was recently detected in the human serum of autoimmune diseases, and it is frequently high in blood and cerebrospinal fluid of MS individuals, who were not subjected to treatment with IFN, and reduced in remission periods [35].

The effects of physical activity in C-reactive protein were evaluated in three studies, showing different results of the effects of physical activity in subjects, with a significant decrease in one study [8], without alterations in the second one [14], and in the third one tending to be lower [22]. Some studies [7, 16] report that during and after moderate-intensive physical activity (70% of VO_2max_, with sprints at 90%) it seems that there were no alterations of CRP. Two other studies suggested that although light intensity training has no influence, there was a decrease in CRP with a moderate/intensive physical activity [3, 36].

The TNF-*α* was evaluated in three studies, with a similar behavior in groups submitted to physical activity and a significant decrease in one study, without changes in a second study, and tending to be lower in the other one [8, 14, 22]. The values of TNF-*α* were similar in groups submitted to physical activity. Recent studies indicate high concentrations of TNF-*α* in MS individuals relating it to the pathogenic process and in MS individuals who do not do physical activity; the TNF-*α* level tends to increase twice to three times and not to increase with exercise [37]. However, for both populations there is a reference to the TNF-*α* decrease in the presence of IL-6.

The influence of IL-6 in cytokines regulation may determine the IL-10 activity, contributing to a decrease in the TNF-*α* concentration and blood-brain barrier permeability. The IL-10 is associated with an inflammation decrease and with the MS remission phases. MS exacerbation symptoms may also result from a low production of IL-10 [38]. Though the baseline levels were significantly lower in MS, it has been suggested that the group of MS submitted to a physical activity presented a response of IL-10 similar to the healthy individuals [14]. This is one of the main anti-inflammatory cytokines able to reduce the blood-brain barrier permeability. In three studies [2, 14, 22] a significant decrease of INF-*γ* was verified in MS groups, and in the healthy individuals there were no significant changes. The presence of IFN-*γ* in the local can control the inflammation, increase IL-10 and sIL-1r, and decrease cellular proliferation. The physical activity effects in this cytokine are not well defined, so there are controversial theories and studies. As some dendritic cell subsets have the capability to activate the Th1 cells that produce IFN-*γ*, the immune dynamic regulation with a protective role in MS [8, 11, 39] can be induced through physical activity.

## 5. Conclusions

Physical activity through physiological mechanisms seems to bestow functional benefits and symptom reduction in MS, without aggravating the inflammatory pathology. During and after physical activity several cytokines are released into the organism, but their influence in the inflammatory process of MS is not yet clear. Aerobic training improves resistance capabilities and the quality of life, while resistance training may have an impact in proinflammatory cytokines, such as TNF-*α* and IFN-*γ*, and in the IL-6 and IL-10 values. Those results are related to the active muscle.

In light physical exercises the cellular activity is lower, while in exercise at 65% of VO_2max⁡_ it seems that there is more capacity to solve an inflammatory situation. The neurologic lesion in MS reveals itself through different motor answers in each individual. So physical activity parameters must be evaluated in order to achieve an individualized physiotherapy intervention program.

Further studies are needed to determine the cytokine concentration with physiologic action in the population with MS, as well as the effect of physical activity to the cytokine balance. In future, studies concerning physical activity and the immunologic activity in MS individuals that usually practice physical activity should continue to be investigated.

## Figures and Tables

**Table 1:** The methodological quality of the studies using the PEDro scale.

Article (first author only)	Eligibility criteria were specified		Subjects were randomly allocated to groups		Allocation was concealed		The groups were similar at baseline		There was blinding of all subjects		There was blinding of therapy administration		There was blinding of all assessors		Outcomes were obtained from more than 85%		At least one key outcome was analyzed by intention to treat		Statistical comparisons between groups		Point measures and measures of variability		Total	
	A	B	A	B	A	B	A	B	A	B	A	B	A	B	A	B	A	B	A	B	A	B	A	B
Castellano 2008 [8]	1	1	1	0	0	0	1	1	0	0	0	1	0	1	1	1	1	1	1	1	1	1	**7**	**8**
Golzari 2010 [2]	1	1	1	1	0	0	1	1	0	0	0	0	0	1	1	1	1	1	1	1	1	1	**7**	**8**
Heesen [14]	1	1	0	0	0	0	1	1	1	1	0	0	1	1	1	1	1	1	1	1	1	1	**8**	**8**
Schulz [12]	1	1	1	1	0	0	1	1	0	0	0	0	0	1	1	1	1	1	1	1	1	1	**7**	**8**
White [22]	1	1	0	0	0	0	1	1	0	0	0	0	1	1	1	1	1	1	1	1	1	1	**7**	**7**

Reviewer 1 (A); reviewer 2 (B).

**Table 2:** Physical activity effects on functional, endocrine, and immunological parameters.

Authors	Physical activity (intensity, frequency, and duration)	Measured cytokines	Sample	Functional and endocrine parameters	Results
Castellano et al., 2011 [8]	30 minutes cycling aerobic exercise 3 times/week for 8 weeks 60% VO_2max_	IL-6 TNF-*α* IFN-*γ*	MS = EG *n* = 11 Healthy = CG *n* = 11 Medication without steroid	VO_2max_; heart rate; EDSS	IL-6: similar between EG and CG (*P* > 0,05). Increased significantly after exercise (*P* = 0,021) TNF-*α*: Tended to be higher in EG before exercise Similar between groups with significant decreases after exercise (*P* < 0,05) IFN-*γ*: increased at rest for MS. Similar for both groups with significant decreases during and after exercise (*P* < 0,05) VO_2max_: significant increase in both groups (*P* < 0,05) EDSS: decrease of disability in MS (3,4–2,6)

Golzari et al., 2010 [2]	30 minutes combined exercise (stretching, aerobic, and endurance exercise) 3 times/week for 8 weeks	IL-4 IL-17 IFN-*γ*	MS *n* = 20 EG = 10 CG = 10 Medication without anti-inflammatory	VO_2max_; heart rate; EDSS	During rest the immunological values were similar IL-4: without changes IL-17: significant decrease in EG (*P* < 0,05) IFN-*γ*: without changes in CG and significant decrease in EG during the training (*P* < 0,05) VO_2max_: without changes EDSS: significant improvement EG (*P* = 0,043)

Heesen et al., 2003 [14]	30 minutes cycling aerobic exercise twice/week for 8 weeks 75% VO_2max_	IL-10 TNF-*α* IFN-*γ*	MS = EG *n* = 15 with training Healthy = CG *n* = 10 MS = CGE *n* = 13 without training Medication without steroid and immunotherapy	VO_2max_; heart rate; EDSS; lactates; endocrines values	IL-10: low during the rest in EG and CGE (*P* = 0,043) increasing in CG e EG (*P* = 0,02) TNF-*α*: without changes in CG and EG (*P* = 16) with decrease in CGE IFN-*γ*: significant decrease in EG (*P* = 0,016) and CG (*P* = 0,01) No significant changes in GCE VO_2max_: significant increase ACTH was low in EG

Schulz et al., 2004 [12]	30 minutes cycling aerobic exercise twice/week for 8 weeks 75% VO_2max_	IL-6 sIL-6r	MS *n* = 28 EG = 15 CG = 13 Medication without steroid Immunotherapy included	VO_2max_; heart rate; EDSS; lactates; endocrines value BDNF; NGF	IL-6: without changes before (*P* = 0,24) or after the activity (*P* = 0,21) sIL-6r: during the rest without changes (*P* = 0, 22); EG Tended to increase during the exercise in EG (*P* = 0,08) without changes in CG VO_2max_: significant increase in EG (*P* < 0,001) EDSS: increase in EG (*P* < 0,05) BDNF and NGF Tended to increase in EG and lower in CG (*P* = 0,18 e 0,12, resp.)

White et al., 2006 [22]	30 minutes cycling endurance twice/week for 8 weeks starting at 40% until 70% VO_2max_	IL-6 IL-10 TNF-*α* IFN-*γ*	MS *n* = 10 Medication without steroid and immunotherapy	VO_2max_; heart rate; CRP; EDSS; fatigue	IL-2 e IL-6: without changes (*P* > 0,05) IL-10: low concentration during rest (both with *P* = 0,011) TNF-*α*: Tended to be lower (*P* = 0,069) IFN-*γ*: at rest there was a lower concentration (*P* = 0,008)—after training CRP: significant decrease (*P* = 0,007) EDSS: significant improvement in all individuals (*P* < 0,05)

EG: experimental group; CG: control group; CGE: MS patients without training; EDSS: expanded disability status scale; ACTH: adrenocorticotropic hormone; BDNF: neutrophils factor; NGF: growth factors; VO_2max⁡_: maximal oxygen consumption; TNF-*α*: tumor necrosis factor-alpha; IFN-*γ*: interferon-gamma; CRP: C-reactive protein.
